# Chorea and Anemia

**Published:** 1904-09

**Authors:** Roshier W. Miller

**Affiliations:** Barton Heights, Va., Lecturer on Nervous and Mental Diseases, and Professor of Theory and Practice of Pharmacy, University College of Medicine, Richmond, Va.


### CHOREA AND ANEMIA.

By ROSHIER W. MILLER, M.D., Ph.G., Barton Heights, Va.,

Lecturer on Nervous and Mental Diseases, and Professor of Theory and Practice of Pharmacy, University College of Medicine, Richmond, Va.

In the etiology of chorea, nothing is noted relative to anemia. It is simply accounted as an accompanying symptom of the condition. Medical literature emphasizes the relation between rheumatism and chorea, with anemia as an important symptom. After observation of several cases, I am strongly of opinion, however, that anemia as a causative factor is worthy of investigation.

Anemia of toxic origin presents pathological conditions which favor the production of choreaic affections. It is true that simple anemia is, as a rule, of secondary origin, and, viewed in this light, it may be argued that if chorea arises, it is the result of the primary and not of the secondary conditions—thus agreeing with the admitted etiology. This argument, however, will not satisfactorily explain those cases of chorea which arise remotely from the primary condition, but recently from the secondary effects.

I submit three cases in which symptoms, treatment and recovery seem to intimate at least a possible relation between anemia and chorea.

CASE 1.—A female child of eight years gave a history of typhoid fever eight months prior to my visit. According to the mother's statement, the child had made a quick and good recovery, gaining rapidly in weight and exhibiting the energy of her former life. Six months later she became irritable and pale, with pain in her arms and legs, which condition was soon followed by gastric disorders and irregular spasms of the muscles of the face. Simple anemia was in evidence from objective and subjective symptoms alone, but was unquestioned in the light of the results obtained from blood examination—the red-blood element being present to the extent of barely 3,000,000 red corpuscles per c. m.

This case was treated with two teaspoonfuls of pepto-mangan

(Gude) and two drops of Fowler's solution, three times a day. After gastric symptoms had abated somewhat, two raw eggs per day were added to the diet. The patient was discharged in five weeks, completely recovered.

CASE 2.—A female child of ten years of age; gave history of malaria (a well-defined case of intermittent fever) one year previously. The pallid condition of the child induced the mother to solicit my aid. Upon examination, I found slight choreaic movements which had escaped the mother's eye, though she did admit that the child "could not sit still very long at a time," and "was constantly working her fingers." The blood examination revealed no plasmodium. The red cells were reduced to 2,800,000 per c. m., with a proportionate decrease of hemoglobin.

Pepto-mangan (Gude) alone was employed in doses of two drams in a glass of milk three times a day. The blood examination four weeks later showed red cells present to the amount of 3,900,000 per c. m., at which time I dismissed the case, completely recovered.

CASE 3.—A female child of thirteen years. Two months before my visit, the mother informed me, the child became peevish and pale, and was reproved at school for her inability to write neatly. She was taken from school, but she grew rapidly worse. Morning nausea, vomiting, headache and anorexia were her daily companions. I found her with pronounced histrionic spasm, with involvement of the upper and lower extremities. Hemic murmurs were plainly apparent, but no endocardial irritation could be determined. The blood count showed reduction in red cells to 2,100,000 per c. m. The hemoglobin was reduced to a degree greater than the red cells. A curious feature of the case was the morning nausea. Immediately upon awakening, she experienced nausea, which was followed by vomiting. I discovered, however, that this condition was superinduced by odors from the kitchen, and directed that a small sponge, moistened with creosote water, be placed over the nose and mouth before the preparation for breakfast began. The annoying symptom was promptly checked by this simple method. The anemia in this case may have been produced by malnutrition, but even this view is mere speculation.

The irritability of the stomach in this case was so pronounced that I did not deem it wise to give nourishment—not to speak of medicine—by the stomach. During the first four days rectal alimentation was employed. A nutritive enema, consisting of four ounces of peptonized milk and two drams of pepto-mangan (Gude), was given every six hours. Small amounts of peptonoids with creosote on ice were given by the stomach. Egg albumin was taken in all the water she drank. After four days, the stomach was tested with small amounts of milk and pepto-mangan (Gude). Beginning with four ounces of milk and one dram of pepto-mangan (Gude) every four hours, the amounts of each were rapidly increased, until after three days the patient was taking eight ounces of milk every two hours and four drams pepto-mangan (Gude) three times a day. This diet, plus three raw eggs a day, together with the above treatment, was all that was employed for six weeks. The blood examination at this time showed a highly gratifying condition—the red cells being present to the extent of 4,100,000 per c. m. The bloom of youth once more tinted the cheek, and the shrine of St. Vitus lost a visitor.—*Virginia Medical Semi-Monthly, May 13, 1904.*

